# Effect of Particle and Substrate Wettability on Evaporation-Driven Assembly of Colloidal Monolayers

**DOI:** 10.1021/acs.langmuir.5c01195

**Published:** 2025-06-04

**Authors:** Qingguang Xie, Tian Du, Christoph J. Brabec, Jens Harting

**Affiliations:** † Helmholtz Institute Erlangen-Nürnberg for Renewable Energy (IET-2), 28334 Forschungszentrum Jülich, Cauerstraße 1, 91058 Erlangen, Germany; ‡ Helmholtz Institute Erlangen-Nürnberg for Renewable Energy (IET-2), 28334 Forschungszentrum Jülich, Immerwahrstraße 2, 91058 Erlangen, Germany; § Institute of Materials for Electronics and Energy Technology (i-MEET), Department of Materials Science and Engineering, 9171 Friedrich-Alexander-Universität Erlangen-Nürnberg, Martensstraße 7, 91058 Erlangen, Germany; ∥ Department of Chemical and Biological Engineering and Department of Physics, 9171 Friedrich-Alexander-Universität Erlangen-Nürnberg, Cauerstraße 1, 91058 Erlangen, Germany

## Abstract

Assembled monolayers of colloidal particles are crucial for various applications, including optoelectronics, surface engineering, as well as light harvesting, and catalysis. A common approach for self-assembly is the drying of a colloidal suspension film on a solid substrate using technologies such as printing and coating. However, this approach often presents challenges such as low surface coverage, stacking faults, and the formation of multiple layers. We numerically investigate the influence of substrate and particle wettability on the deposited pattern. Higher substrate wettability results in a monolayer with a hexagonal arrangement of deposited particles on the substrate. Conversely, lower substrate wettability leads to droplet formation after the film ruptures, leading to the formation of particle clusters. Furthermore, we reveal that higher particle wettability can mitigate the impact of substrate wettability and facilitate the formation of highly ordered monolayers. We propose theoretical models predicting the surface coverage fraction dependent on particle volume fraction, initial film thickness, particle radius, as well as substrate and particle wettability, and validate these models with simulations. Our findings provide valuable insights for optimizing the deposition process in the creation of assembled monolayers of colloidal particles.

## Graphical abstract

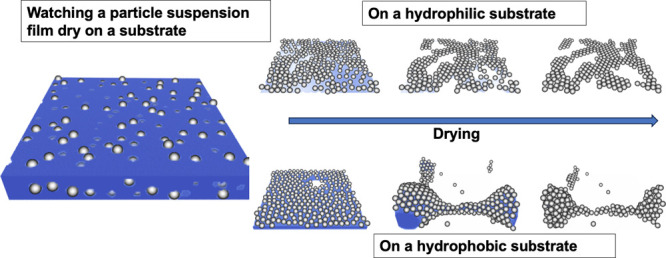

## Introduction

The assembly of monolayers of colloidal particles is relevant in various scientific and technological domains, namely catalysis, photovoltaics, sensors, nanomedicine and batteries.^1–6^ Self-assembled monolayers are used as a mask to fabricate ordered nanostructures in colloidal lithography.^7^ Moreover, self-assembled monolayers can significantly influence the properties of the overall structure. For example, solution-processed thin films based on metal oxide nanoparticles were widely employed in organic or hybrid optoelectronic devices, as they are the particularly versatile materials used as interfacial buffer layers to resolve energetic misalignment in organic electronics.^8,9^ Their applications range from charge injection layers in light-emitting diodes, gate layers in organic field-effect transistors, charge extraction layers for organic solar cells^10^ to more recently charge-transporting layers in perovskite solar cells.^11^ Additionally, broadband light absorption enhancement has been observed in ultrathin film crystalline silicon solar cells with the incorporation of polystyrene colloidal monolayers.^12^

The assembly of monolayers of colloidal particles is usually done by drying a suspension film on a substrate.^13–15^ Here, one utilizes techniques such as printing and coating, which are easy-to-use, low-cost and scalable.^16,17^ Furthermore, the flat fluid–fluid interface prevents capillary flow and radial movement of particles, usually encountered in drying a colloidal suspension droplet due to contact line pinning.^18,19^ However, achieving a uniform deposition pattern from drying a thin film of colloidal particles also poses formidable challenges due to several inherent complexities. The deposition of particles is susceptible to interparticle forces such as van der Waals attraction and capillary forces, as well as particle-fluid and particle-substrate interactions.^3,20,21^ Zargartalebi et al.^22^ produced highly ordered particle deposits by drying a suspension film on a superhydrophilic substrate surrounded by a neutrally wetting mold with low roughness. They claimed that a meniscus-free interface and a hydrophilic substrate are required to produce highly ordered particle assemblies. Fujita et al.^23^ numerically addressed the effect of particle wettability on the deposition process on a hydrophilic substrate. Similarly, Mino et al.^24^ simulated the drying process of a colloidal suspension on a wetting substrate. Their findings revealed that particles with higher wettability exhibited slower aggregation. However, the effect of substrate wettability and its interplay with particle wettability on the deposited pattern are neglected, despite their pivotal roles in determining the process of particle deposition.

In this paper, we perform simulations of drying a colloidal suspension film utilizing a coupled lattice Boltzmann and discrete element method. The lattice Boltzmann method is a powerful tool to model fluid flow involving solvent evaporation.^25^ The particles are discretized on the lattice and are coupled with a fluid solver through a momentum exchange approach.^26,27^ Initially, we compare the temporal evolution of the evaporated mass during the drying process of both a pure liquid film and a colloidal suspension film on a substrate with its respective analytical prediction. Subsequently, we explore the particle deposition resulting from the drying of a colloidal suspension film, manipulating the substrate wettability. On a well wetting substrate, the film undergoes drying and dewetting, resulting in the formation of a monolayer deposit during the evaporation process. Conversely, lower substrate wettability leads to film rupture and droplet formation, leaving behind particle clusters after drying. Importantly, our findings furthermore demonstrate that the particle wettability has the capability to mitigate the influence of the substrate wettability. We propose theoretical models to predict the surface coverage fraction, considering the particle volume fraction and incorporating the wetting properties of both particle and substrate. These models are in good agreement with our simulation results.

## Methods

We employ the lattice Boltzmann method (LBM), a computational technique used for modeling fluid dynamics at the mesoscopic scale, offering a unique and versatile approach to simulate complex fluid flow phenomena.^28^ Unlike traditional methods based on solving the Navier–Stokes equations directly, the LBM is rooted in kinetic theory, employing a lattice to represent fluid particles and their collisions. In the regime of small Knudsen and Mach numbers, the Navier–Stokes equations are reinstated.^28^ Over the last two decades, the LBM has proven itself as a robust tool for numerically simulating fluid flows.^28^ It has been expanded to model multiphase/multicomponent fluids^29,30^ and suspensions of particles with varying shape and wettability.^26,27,31,32^ The inherent parallelizability and adaptability of the LBM to irregular geometries make it particularly advantageous for studying intricate fluid dynamics scenarios. In the subsequent discussion, we outline relevant details and direct readers to the relevant literature for an in-depth description of the method and our implementation.^25,27,30,33^

We utilize the pseudopotential multicomponent LBM of Shan and Chen^29^ with a D3Q19 lattice.^34^ Here, two fluid components are modeled by following the evolution of each distribution function discretized in space and time according to the lattice Boltzmann equation,

$$\begin{array}{rllllll} f_{i}^{c}(x+e_{i}\Delta t,t+\Delta t) & =f_{i}^{c}(x,t)-\frac{\Delta t}{\tau^{c}}[f_{i}^{c}(x,t) & & & & & \\ & -f_{i}^{\mathrm{eq}}(\rho^{c}(x,t),u^{c}(x,t))] & & & & & \end{array}$$ 1

where *i* = 0, ···, 18. *f*_*i*_^*c*^(**x**, *t*) are the single-particle distribution functions for fluid component *c* = 1 or 2, and **e**_*i*_ is the discrete velocity in the *i*th direction. τ^*c*^ is the relaxation time for component *c* and determines the viscosity. The macroscopic densities and velocities for each component are defined as ρ^*c*^(**x**, *t*) = ρ_0_ ∑_*i*_ *f*_*i*_^*c*^(**x**, *t*), where ρ_0_ is a reference density, and **u** ^*c*^(**x**, *t*) = ∑_*i*_ *f*_*i*_^*c*^(**x**, *t*)**e**_*i*_/ρ^*c*^(**x**, *t*), respectively. Here, *f*_*i*_^eq^ is the second-order equilibrium distribution function defined as

$$\begin{array}{ccc} f_{i}^{\mathrm{eq}}(\rho^{c},u^{c}) & = & \omega_{i}\rho^{c}[1+\frac{e_{i}\cdot u^{c}}{c_{s}^{2}}-\frac{\left(u^{c}\cdot u^{c}\right)}{2c_{s}^{2}}+\frac{(e_{i}\cdot u^{c}{)}^{2}}{2c_{s}^{4}}] \end{array}$$ 2

where ω_*i*_ is a coefficient depending on the direction: ω_0_ = 1/3 for the zero velocity, ω_1_,...,_6_ = 1/18 for the six nearest neighbors and ω_7_,...,_18_ = 1/36 for the nearest neighbors in diagonal direction. $c_{s}=\frac{1}{\sqrt{3}}\frac{\Delta x}{\Delta t}$ is the speed of sound.

For convenience, we choose the lattice constant Δ*x*, the time step Δ*t*, the reference density ρ_0_ and the relaxation time τ^*c*^ to be unity, which leads to a kinematic viscosity $\nu^{c}=\frac{1}{6}$ in lattice units.

The pseudopotential multicomponent model introduces a mean-field interaction force

$$F^{c}\left(x,t\right)=-\Psi^{c}\left(x,t\right)\underset{\overset{®}{c}}{\sum }\underset{i}{\sum }\omega_{i}g_{c\mathrm{c̅}}\Psi^{\mathrm{c̅}}\left(x+e_{i},t\right)e_{i}$$ 3

between fluid components *c* and *c̅*,^29^ in which *g*_*cc̅*_ is a coupling constant, eventually leading to a demixing of the fluids. We denote γ as the surface tension of the interface. Ψ^*c*^(**x**, *t*) is an “effective mass”, chosen as the functional form

$$\Psi^{c}\left(x,t\right)≡\Psi \left(\rho^{c}\left(x,t\right)\right)=1-e^{-\rho^{c}\left(x,t\right)}$$ 4

This force **F** ^*c*^(**x**, *t*) is then applied to the component *c* by adding a shift $\Delta u^{c}\left(x,t\right)=\frac{\tau^{c}F^{c}\left(x,t\right)}{\rho^{c}\left(x,t\right)}$ to the velocity **u** ^*c*^(**x**, *t*) in the equilibrium distribution.

When the interaction parameter *g*_*cc̅*_ in eq 3 is appropriately selected, the separation of components occurs, leading to the formation of distinct phases. Each component segregates into a denser majority phase with a density of ρ_*ma*_ and a lighter minority phase with a density of ρ_*mi*_. The diffusive nature of the interface prevents the occurrence of stress singularities at the moving contact line, a phenomenon typically observed in sharp-interface models.

To model substrate wettability, we introduce an interaction force between the fluid and wall, inspired by the work of Huang et al.,^35^ as

$$F^{c}\left(x\right)=-g^{wc}\Psi^{c}\left(x\right)\underset{i}{\sum }\omega_{i}s\left(x+e_{i}\right)e_{i}$$ 5

where *g* ^*wc*^ is a constant. Here, *s*(**x** + **e**_*i*_) = 1 if **x** + **e**_*i*_ is a solid lattice site, and *s*(**x** + **e**_*i*_) = 0 otherwise.

To induce evaporation, we enforce a constant value ρ_*H*_^*c*^ for the density of component *c* at the boundary sites **z**_*H*_ by specifying the distribution function of component *c* as^25^

$$f_{i}^{c}\left(z_{H},t\right)=f_{i}^{\mathrm{eq}}\left(\rho_{H}^{c},u_{H}^{c}\left(z_{H},t\right)\right)$$ 6

Here, **u**_*H*_^*c*^(**z**_*H*_, *t*) = 0. If the prescribed density ρ_*H*_^*c*^ is lower than the equilibrium minority density ρ_*mi*_^*c*^, a density gradient is established in the vapor phase of component *c*. This gradient prompts the diffusion of component *c* toward the evaporation boundary. The diffusion coefficient of component *c* is given as $D_{c}=c_{s}^{2}\left(\tau -\frac{1}{2}\right)\frac{\rho_{\mathrm{c̅}}}{\rho_{c}+\rho_{\mathrm{c̅}}}-\frac{c_{s}^{2}\rho_{c}g_{\mathrm{c̅}c}\Psi_{c}^{\prime }\Psi_{\mathrm{c̅}}}{\rho_{c}+\rho_{\mathrm{c̅}}}$ , where Ψ′ = dΨ/dρ.^25,36^ It is important to note that our evaporation model is diffusion-dominated, which is validated in our prior work.^25^

The colloidal particles are discretized on the fluid lattice, and their interaction with the fluid species is established through a modified bounce-back boundary condition, a method pioneered by Ladd and Aidun.^26,37^ The motion of the particles is governed by classical equations of motion:

$$Fp=m\frac{du_{p}}{dt}$$ 7

Here, **F**p represents the total force acting on a particle with mass *m*, and **u**_p_ is the particle’s velocity. The trajectory of a colloidal particle is updated using a leapfrog integrator. Given that we treat particles as rigid spheres, we neglect rotational motion and particle deformation.

We introduce a “virtual” fluid within the outer shell of the particle, with an amount Δρ_*p*_,^27,33^ expressed as

$$\rho_{\mathrm{virt}}^{1}(x,t)={\overset{®}{\rho }}^{1}(x,t)+\Delta \rho_{p}$$ 8

$$\rho_{\mathrm{virt}}^{2}(x,t)={\overset{-}{\rho }}^{2}(x,t)-\Delta \rho_{p}$$ 9

ρ̅^1^(**x**, *t*) and ρ̅^2^(**x**, *t*) represent the averages of the density of neighboring fluid nodes for components 1 and 2, respectively. The virtual fluid inside the particles is incorporated into the calculation of the Shan-Chen interaction force eq 3, which ensures a proper force balance and prevents the formation of an artificial fluid density layer around the particles.^27^ The Shan-Chen interaction between the particles and the surrounding fluids can be tuned by adjusting the density of the local virtual fluid. Increasing the density of one component by an amount Δρ_*p*_ makes the particle surface “prefer” that fluid over the other. The parameter Δρ_*p*_, referred to as the “particle color”, governs the particle’s wettability and thus determines its contact angle. The contact angle varies approximately linearly with the particle color, with the slope of this relationship depending on the particular simulation parameters used.^27^ A particle color of Δρ_*p*_ = 0 corresponds to a contact angle of θ = 90°, indicating a neutrally wetting particle.

The exchange of momentum between particles and the fluid accounts for hydrodynamic forces, including drag and lift forces. Our model accurately captures lubrication interactions when the distance between two particles is at least one lattice site. However, when the separation is less than one lattice site, a lubrication correction is applied^26,38,39^ :

$$F_{ij}=-\frac{3\pi \mu R^{2}}{2}{\mathrm{r̂}}_{ij}{\mathrm{r̂}}_{ij}\cdot \left(u_{i}-u_{j}\right)\left(\frac{1}{r_{ij}-2R}-\frac{1}{\Delta_{c}}\right)$$ 10

Here, *R* represents the radius of the particle, ${\hat{r}}_{ij}=\frac{r_{i}-r_{j}}{\left|r_{i}-r_{j}\right|}$ is a unit vector pointing from the center of one particle to the center of the other, and *r*_*ij*_ is the distance between particles *i* and *j*. The velocities of particles are denoted by **u**_*i*_ and **u**_*j*_. The constant Δ_*c*_ is chosen as Δ_*c*_ = 2/3.

The van der Waals forces acting between two spherical particles with identical radii *R* are modeled as

$$F_{\mathrm{vdW}}=\frac{A_{H}R}{12(r_{ij}-2R{)}^{2}},\;\mathrm{for}\;r_{s}\leq r_{ij}\leq r_{c}$$ 11

where *A*_*H*_ is the Hamaker constant, and *r*_*s*_ and *r*_*c*_ are the cut off radii. We set *r*_*s*_ = 2*R* + 0.2 and *r*_*c*_ = 2*R* + 1 in our simulations.

To prevent the overlap of particles, we introduce a Hertz potential:^40^

$$\phi_{H}=\{\begin{array}{l} K_{H}(2R-r_{ij}{)}^{5/2}\;\mathrm{for}\;r_{ij}\leq 2R\\ 0,\mathrm{otherwise} \end{array}$$ 12

Here, *K*_*H*_ is the force constant and is chosen to be *K*_*H*_ = 100. The Hertz hard-sphere potential governs particle–particle interactions at close contact, eliminating the need for an explicit contact model.

For the interactions between particles and a substrate, the lubrication forces between particles and walls are modeled similarly to the lubrication forces between particles themselves. Additionally, to prevent particle-substrate penetration and model particle adsorption onto the substrate, we implement the Lennard-Jones (LJ) potential between particles and a substrate as

$$\phi_{\mathrm{LJ}}=4\epsilon \left[{\left(\frac{\sigma }{r_{iw}}\right)}^{12}-{\left(\frac{\sigma }{r_{iw}}\right)}^{6}\right]\;\;\mathrm{for}\;r_{iw}\leq r_{c2}$$ 13

where ϵ is the depth of the potential well, σ is the finite distance at which the interparticle potential is zero, *r*_*iw*_ is the distance between a particle center and the substrate surface and *r*_*c*2_ is the cut off radius. We set σ equal to the particle radius *R* and *r*_*c*2_ = 2.5*R* in all simulations.

Our numerical models were validated previously by comparing the capillary forces between neighboring particles at fluid interfaces,^32,41^ the evolution of interface position when drying a purely liquid film and a floating droplet,^25^ as well as the velocity field in an evaporating sessile droplet with theoretical analysis and experimental observations.^42^ We note that the evaporation-driven dynamics during the drying of a colloidal film are primarily governed by vapor diffusion through the surrounding fluid phase, whereas the specific properties of the surrounding fluid have a negligible influence on the overall behavior. Therefore, we employed a multicomponent model instead of a multiphase model to ensure numerical stability.

The parameter values chosen in our simulations correspond to particles with a radius on the order of 100 *nm* in water, which has a dynamic viscosity of η_*w*_ = 10^–3^ Pa · s, a mass density of ρ_*w*_ = 10^3^ kg/m^3^, and a surface tension of σ_*w*_ = 7.2 × 10^–2^ N/m. We consider a system in which particle diffusion is much slower than the movement of the liquid interface driven by evaporation, implying a fast-evaporation regime characterized by a high Péclet number, *Pe* ≫ 1, defined as the ratio of the characteristic time scales of particle diffusion and interface movement. Consequently, the Brownian motion of colloidal particles is neglected in our simulations. The droplet shape is dominated by surface tension, corresponding to a small Bond number (*Bo* ≪ 1), such that gravitational effects are negligible. Furthermore, we assume the particles have a density similar to that of the liquid, so gravitational forces are not applied to the particles.

## Results and Discussion

We investigate the evaporation of a planar film on a solid substrate, as illustrated in Figure 1 and perform simulations with a system size of 128 × 128 × 128 lattice nodes, unless specified otherwise. One portion of the system is filled with fluid *c*, while the other contains an equally dense fluid *c̅*. This setup results in the emergence of a fluid–fluid interface at position *z*_0_. The position of the interface is defined as the position where ρ_*c*_ = ρ_*c̅*_. For the interaction between the fluids, we choose a strength of *g*_*cc̅*_ = 3.6 in eq 3, yielding a diffusive interface with a thickness of ≈5 lattice nodes and a corresponding surface tension γ ≈ 0.47. A wall with a thickness of 2 lattice nodes is positioned at the bottom, parallel to the interface, and is enforced with simple bounce-back boundary conditions. The boundaries perpendicular to the substrate are set to be periodic. The van der Waals force between particles is applied, as described by eq 11 with *A*_*H*_ = 0.0467. A Lennard-Jones potential is employed between particles and the substrate, following eq 13. We note that the friction force between the particles and the substrate can significantly influence particle deposition. However, in this context, we assume zero friction between the particle and the substrate, given the dominance of capillary forces.^42^

**Figure 1:**
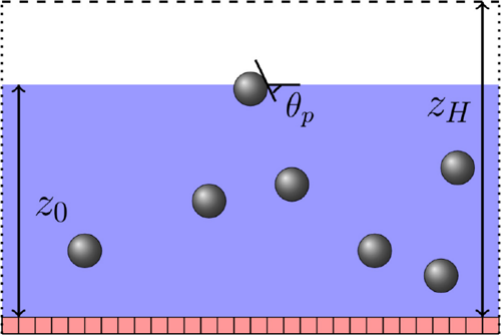
Illustration of a thin colloidal suspension film at the initial state. The initial height of the film is *z*_0_, and the contact angle of the particle is θ_*p*_. We place a substrate with defined wettability at the bottom, whereas the boundaries normal to the substrate are periodic (dotted lines). After equilibration, we apply evaporation boundary conditions at the top of the system (shown by the dashed line). The distance between the evaporation boundary and the substrate is *z*_*H*_ .

### Drying Dynamics of a Film

We start with investigating the drying dynamics of both, a pure liquid film and a colloidal suspension film. The contact angle of the substrate is fixed at θ_*s*_ = 90° and after allowing the system to equilibrate without evaporation, we impose the evaporation boundary condition and monitor the evaporated mass over time.

Figure 2 illustrates the temporal evolution of the evaporated mass for both, a pure liquid film (triangles) and a colloidal suspension film, considering various particle volume fractions *c*_*v*_ = 0.008 (circles), *c*_*v*_ = 0.044 (squares), and *c*_*v*_ = 0.061 (pentagons). We use particles with a radius of *R* = 6 lattice nodes (corresponding approximately to the order of 100 nm) to eliminate the effects of the diffusive interface, such that the particles effectively cover the interface rather than behaving as if they are immersed within it. Initially, the particles are randomly dispersed in the liquid. As drying progresses, an increasing number of particles accumulates at the interface, with the maximum interface coverage fraction ranging from 9.7% to 70%, depending on the selected volume fractions.

**Figure 2:**
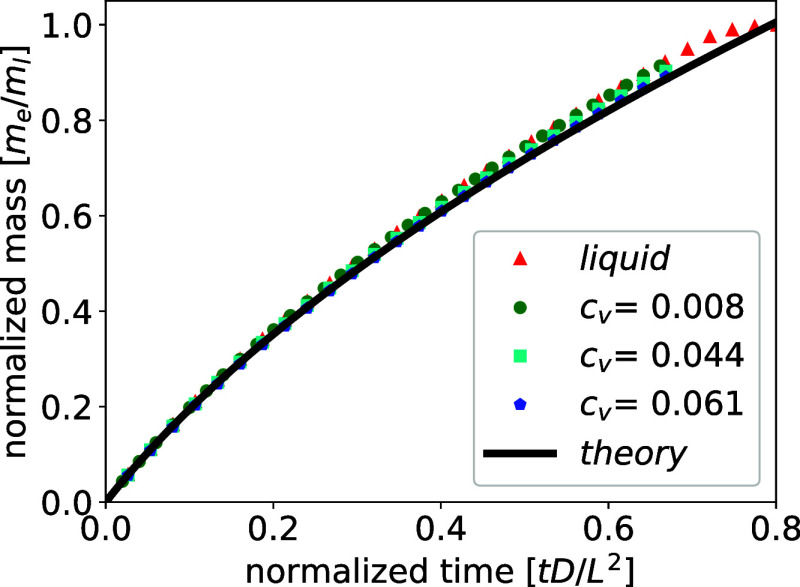
Time evolution of evaporated mass from a drying colloidal suspension film for particle volume fractions *c*_*v*_ = 0.008 (circles), *c*_*v*_ = 0.044 (squares), and *c*_*v*_ = 0.061 (pentagons), in comparison with evaporating a pure liquid film (triangles) and the theoretical prediction eq 21 (solid line). The evaporated mass *m*_*e*_ is normalized by the initial total mass *m*_*l*_ and the time is normalized by the diffusivity *D* of the liquid and the size of the system *L*.

The evaporated mass is normalized as *m*_*e*_/*m*_*l*_, where *m*_*l*_ = ρ_*c*_ *L* ^2^ *z*_0_ is the initial total mass of liquid in the case of a purely liquid film. The time is normalized with the diffusivity *D* ≈ 0.117 of the fluid, and the length *L* = 128 of the system. While the presence of particles at interfaces is expected to influence liquid evaporation, we surprisingly observe overlapping curves across all cases. In the following, we present a theoretical analysis to elucidate the observed phenomena.

By assuming the formation of a linear density gradient in the vapor phase, the evaporation flux can be estimated as^25^

$$j=-D\nabla \rho =-D\frac{\rho_{H}-\rho_{mi}}{z_{H}-z_{i}}n$$ 14

where **n** is the normal vector of the interface. The time derivative of the mass of the liquid is

$$\frac{dm}{dt}=A|j|$$ 15

in which *A* is the area of the interface. In the case that the thickness of the diffusive interface is significantly smaller than the system size, the total mass of the system is approximately

$$m=A\left[z_{i}\rho_{ma}+\left(z_{H}-z_{i}\right)\left(\rho_{mi}-\rho_{H}\right)/2\right]$$ 16

and we obtain

$$\frac{dm}{dt}=A[\rho_{ma}-(\rho_{mi}-\rho_{H})/2]\frac{dz_{i}}{dt}$$ 17

By comparing eqs 15 and 17,

$$A[\rho_{ma}-(\rho_{mi}-\rho_{H})/2]\frac{dz_{i}}{dt}=-AD\frac{\rho_{H}-\rho_{mi}}{z_{H}-z_{i}}$$ 18

we obtain the evolution of the interface position *z*_*i*_ as

$$\frac{dz_{i}}{dt}=\frac{D(\rho_{H}-\rho_{mi})}{\left(z_{H}-z_{i})[\rho_{ma}-(\rho_{mi}-\rho_{H})/2\right]}$$ 19

The position of the interface *z*_*i*_ follows

$$z_{i}=z_{H}-{\left[{\left(z_{H}-z_{0}\right)}^{2}+2\frac{D\left(\rho_{mi}-\rho_{H}\right)}{\rho_{ma}-\frac{\rho_{mi}-\rho_{H}}{2}}t\right]}^{1/2}$$ 20

and the evaporated mass *m*_*e*_ is

$$m_{e}=A\rho_{ma}\left(z_{0}-z_{i}\right)=A\rho_{ma}\left(z_{0}-z_{H}\right)+A\rho_{ma}{\left[{\left(z_{H}-z_{0}\right)}^{2}+\frac{2D\left(\rho_{mi}-\rho_{H}\right)}{\rho_{ma}-\frac{\rho_{mi}-\rho_{H}}{2}}t\right]}^{1/2}$$ 21

Figure 2 shows that the analytical prediction eq 21 (solid line) agrees well with simulation results (symbols).

We note that the evaporated mass can be written in an alternative form as

$$m_{e}=\int_{0}^{t}A|j|dt$$ 22

Traditionally, the interface area *A* is considered the effective evaporation area. However, using this approach would imply a slowdown in evaporation with an increasing particle volume fraction, as particles occupy a portion of the interface. However, if the liquid passes the interface much faster than it would through pure diffusion and if the particle radius is much smaller than the system size, *R* ≪ *z*_*H*_, the vapor phase just above the particles saturates immediately and the evaporation flux remains constant. Consequently, the effective evaporation area remains constant, even in the presence of particles at the interface. As is commonly encountered in the printing and coating processes of catalyst inks or solutions of functional materials used in organic or perovskite solar cells, *z*_*H*_ can be estimated from the initial wet film thickness, which typically ranges from a few tens to hundreds of micrometers—much larger than the particle size, and well within the scope of our proposed model. Furthermore, our findings offer a possible explanation that the theoretical analysis of the velocity field, derived from the drying of a pure liquid droplet, successfully predicts the velocity profile within a drying colloidal suspension droplet.^43–45^

### Effect of Substrate Wettability

In the following, we study the impact of the substrate wettability on the deposition process. We characterize the wettability of the substrate by the contact angle of a droplet on the substrate: a lower contact angle indicates higher wettability, while a higher contact angle corresponds to lower wettability. We initiate the film with a particle volume concentration *c*_*v*_ and choose particles with a radius of 3 to ensure an adequate number of particles while saving computational time. Since our focus is on the effect of substrate contact angle here, the particle radius does not significantly impact the results. The particles are neutrally wetting (contact angle θ_*p*_ = 90°) and we vary the substrate contact angle, examining cases with θ_*s*_ = 30°, θ_*s*_ = 90° and θ_*s*_ = 150°.

In Figure 3, we compare the surface coverage fraction ϕ as a function of particle volume fraction for the different substrate contact angles. To save computational cost, we limit simulations to systems with particle volume fraction ϕ < 0.25. The surface coverage fraction, ϕ, is calculated after the solvent has evaporated, based on the number of particles, *N*_*p*_^*s*^, attached to the substrate using the formula $\phi =\frac{\pi N_{p}^{s}R^{2}}{S}$ (*S* corresponds to the area of the substrate). In all cases, the surface coverage fraction increases with increasing particle volume fraction. At lower volume fraction *c*_*v*_ < 0.08, the surface coverage fraction with a substrate contact angle θ_*s*_ = 30° is slightly higher than that with a contact angle θ_*s*_ = 90°, but the curves overlap at higher volume fraction *c*_*v*_ > 0.1. Throughout the entire range, the surface coverage fraction at contact angles θ_*s*_ = 30° and θ_*s*_ = 90° is larger than that with a contact angle θ_*s*_ = 150°.

**Figure 3:**
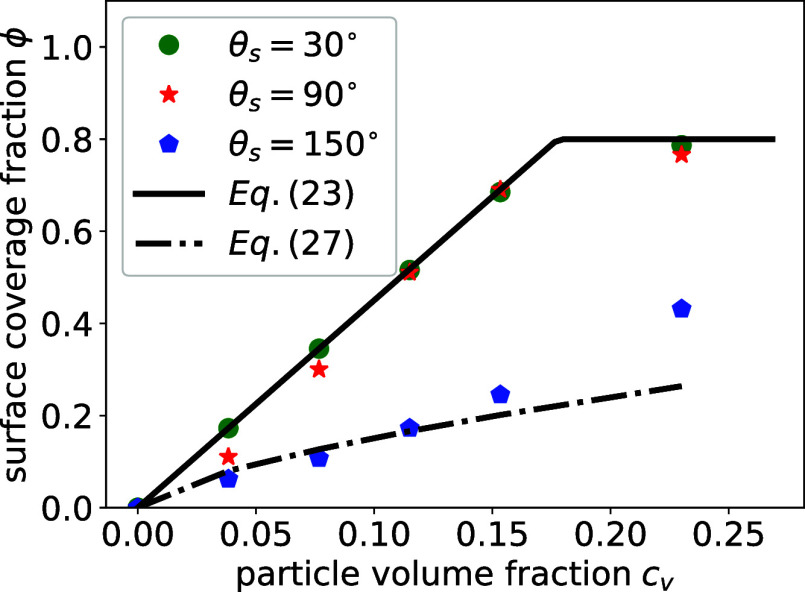
Surface coverage fraction ϕ as a function of particle volume fraction *c*_*v*_ for a substrate with different contact angles θ_*s*_ = 30°, θ_*s*_ = 90°, and θ_*s*_ = 150°. The contact angle of particles is fixed to θ_*p*_ = 90°.

To understand the behavior of surface coverage fraction, in Figure 4 we show snapshots of the drying colloidal suspension film on a substrate with contact angle θ_*s*_ = 30° (Figure 4a–e) and θ_*s*_ = 150° (Figure 4f–j), respectively. The particle volume fraction is *c*_*v*_ = 0.15 and the initial height of the film is *z*_0_ = 30. Initially, the particles are randomly distributed in the liquid or at the interface, as shown in Figure 4a,f. As the drying starts, more particles get attached at the interface (Figure 4b,g). As the interface descends, the particles that get attached to the substrate protrude the interface and deform it. Moving forward, menisci form around the particles, giving rise to capillary forces and resulting in particle aggregation.^13,46^ The aggregation of particles creates voids, ultimately leading to the rupture and dewetting of the film, as the contact line is pinned on the particle surface (Figure 4c,h). At a lower substrate contact angle, dewetting leads to further particle aggregation (Figure 4d). Subsequently, complete evaporation of the liquid occurs, leaving a deposited monolayer on the substrate (Figure 4e). The particles align in a hexagonal arrangement, surrounded by areas of free particles, which is consistent with experimental observations.^13,14,47,48^ At a higher substrate contact angle, after rupture of the film, the liquid film undergoes a retraction process, rapidly forming a droplet (Figure 4i), due to the strong repulsion between the liquid and the substrate. The diffuse interface method employed here inherently accommodates topological changes in the interfacial morphology, effectively avoiding sharp curvature singularities at the rupture point. We note that in our simulations the time scale for the film retraction to the formation of droplets is significantly shorter than the time scale of evaporation. Otherwise, the film may completely dry before forming a droplet. This behavior is consistent with that of a microscale droplet. Considering a droplet with a radius *R*_*d*_ = 1 μm, the characteristic time scale of retraction is $t_{r}=\sqrt{\rho_{w}R_{d}^{3}/\sigma_{w}}\approx 10^{-7}s$ , which is much shorter than the characteristic evaporation time scale *t*_*e*_ = ρ_*w*_ *R*_*d*_^2^/(*D*_*w*_Δχ) ≈ 10^–3^ s. Here, *D*_*w*_ = 2.4 × 10^–5^ m^2^/s is the diffusion coefficient of water vapor in air, and Δχ = 1.2 × 10^–2^ kg/m^3^ is the vapor concentration difference between the surface of the drop and the surroundings. As the film retracts, it entrains and carries particles along, facilitating their migration onto the substrate. Subsequently, particle clusters are deposited on the substrate, as depicted in Figure 4j. The formation of droplets causes particle clustering, which likely explains the disordered arrangement of particles observed when a droplet of an aqueous suspension of monodisperse latex particles dries on hydrophobic substrates.^47^

**Figure 4:**
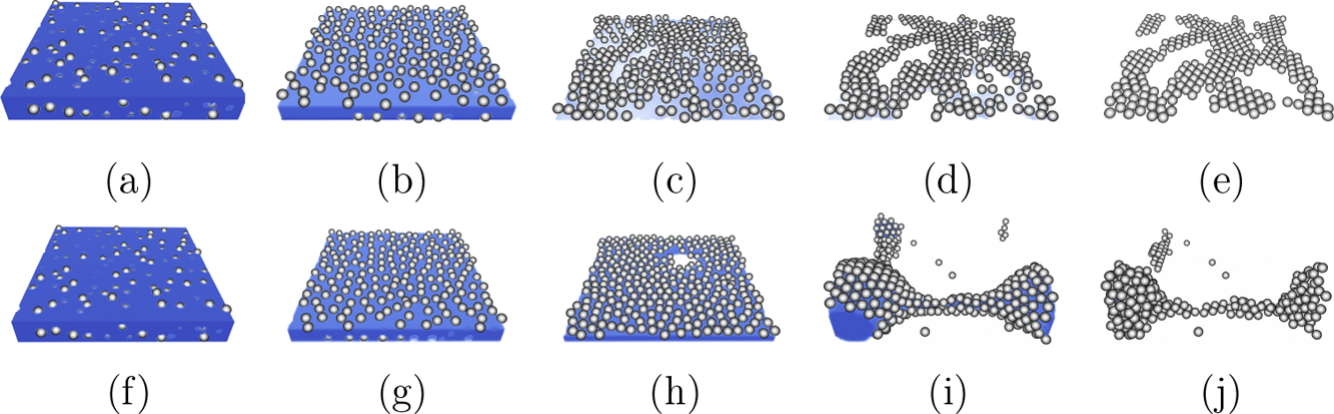
Snapshots of the drying process on a substrate with contact angles θ_*s*_ = 30° (a–e) and θ_*s*_ = 150° (f–j). The particle volume fraction is *c*_*v*_ = 0.15. The fluid is represented in blue color and the particles in gray. For clarity, we omit to show the substrate. At a lower contact angle, the solvent dries and dewets resulting in capillary forces between particles, dragging the particles to form a monolayer. At a higher contact angle, droplets form after film rupture and particle clusters are left after drying.

Figure 5 shows the deposition pattern at different particle volume fractions *c*_*v*_ = 0.04, *c*_*v*_ = 0.08, *c*_*v*_ = 0.15 and *c*_*v*_ = 0.23, on a substrate with a contact angle θ_*s*_ = 30° (Figure 5a–d) and θ_*s*_ = 150° (Figure 5e–h). With a lower substrate contact angle, the particles form monolayers after drying (Figure 5a–c) when the particle volume fraction is low or intermediate. With a higher volume fraction *c*_*v*_ = 0.23, the surface coverage reaches the maximal 2D packing fraction, ϕ ≈ 0.77, and additional particles can be found on top of the first deposition layer (Figure 5d), which is also observed in experiments with higher particle volume fractions.^13,14^ In the case of a higher substrate contact angle, the film retracts after the rupture, forms droplets for low volume fractions, and leaves isolated particle clusters behind (Figure 5e,f). At higher volume fractions, finite size effects may cause the aggregates to form connected clusters (Figure 5g,h).

**Figure 5:**
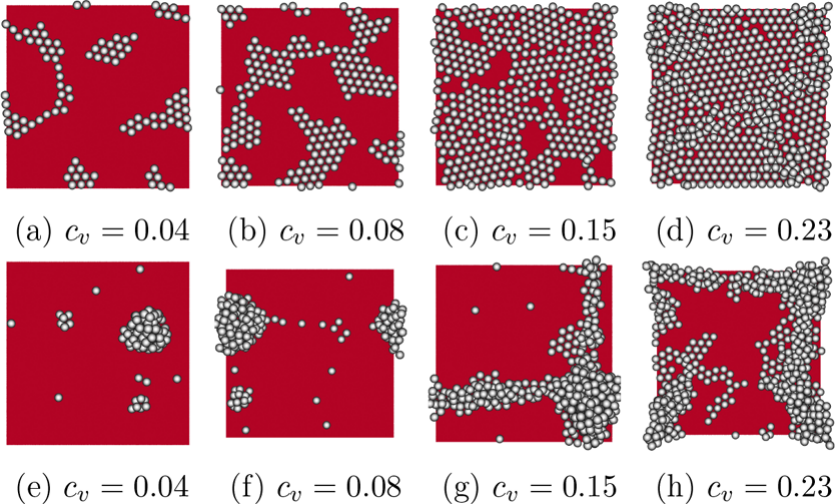
Snapshots of final deposition patterns on a substrate (shown in red) with contact angles θ = 30° (a–d) and θ = 150° (e–g) for different particle volume fractions *c*_*v*_ = 0.04, *c*_*v*_ = 0.08, *c*_*v*_ = 0.15, and *c*_*v*_ = 0.23

### Effect of Particle Wettability

Next, we investigate the effect of the particle wettability, characterized by the particle contact angle at the fluid–fluid interface, on the deposition process and the deposited pattern. The contact angle of the particles is expected to affect the pinning position of the contact line at the particle surface. For the following simulations, we employ again larger particles with a radius *R* = 6 to suppress finite-size effects induced by the diffusive interface.

We perform simulations of a drying colloidal suspension film on a substrate and compare the surface coverage fraction as a function of particle volume fraction with a lower particle contact angle θ_*p*_ = 46°, for different substrate contact angles θ_*s*_ = 30° (circles), θ_*s*_ = 90° (stars) and θ_*s*_ = 150° (pentagons) (see Figure 6a). Here, we simulate systems with higher particle volume fractions, up to ϕ = 0.5, compared to those shown in Figure 3. Due to the larger particle size (*R* = 6 lattice nodes), the total number of particles was reduced by approximately a factor of 8 relative to systems with the same volume fraction but smaller particles (*R* = 3 lattice nodes). As a result, the computational cost was significantly lower. Different from the case shown in Figure 3, where the surface coverage fraction behaves quite differently with neutral particles (θ_*p*_ = 90°), here the surface coverage fraction is similar for different substrate contact angles when the particles have a lower contact angle. Additionally, we performed simulations using particles and a substrate with higher contact angles of θ_*p*_ = 108° and θ_*s*_ = 150°, respectively. The resulting surface coverage fraction for particles with θ_*p*_ = 108° (represented by squares in Figure 6a) is significantly lower than that obtained with particles having a lower contact angle of θ_*p*_ = 46° (represented by pentagons in Figure 6a).

**Figure 6:**
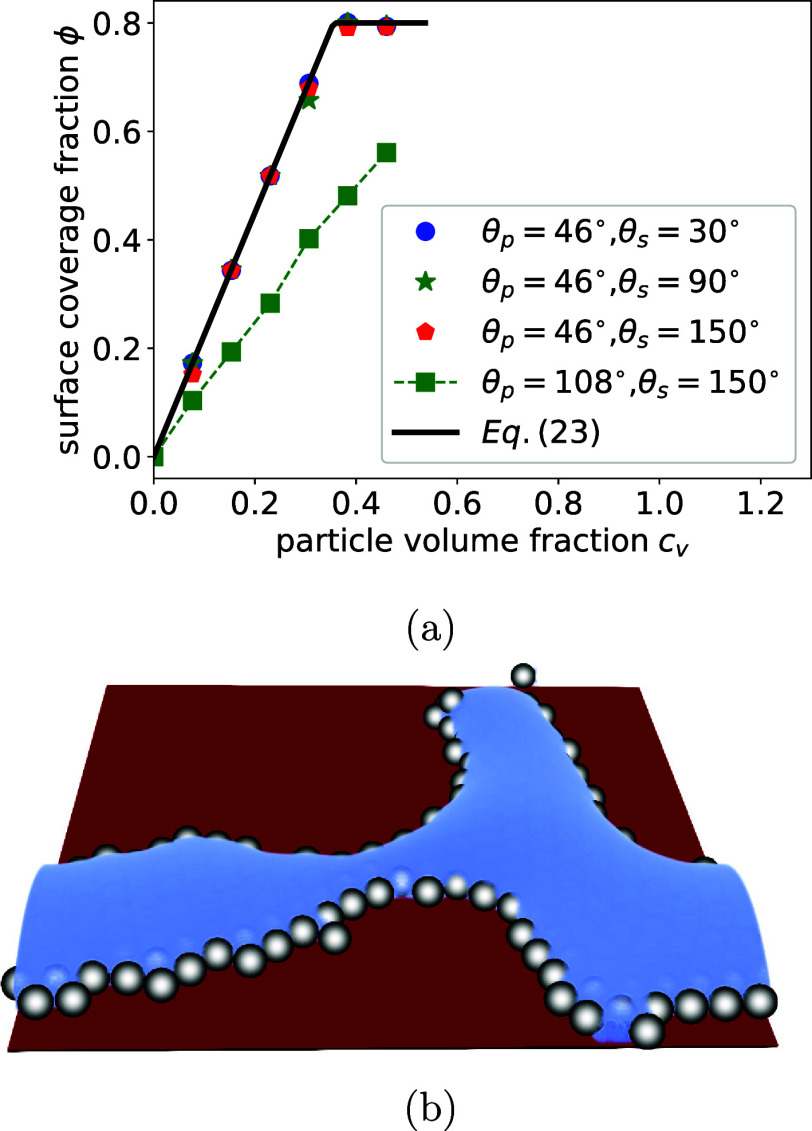
(a) Surface coverage fraction as particle volume fraction *c*_*v*_ for different substrate contact angles θ_*s*_ = 30°, θ_*s*_ = 90°, θ_*s*_ = 150°. The radius of the spherical particles is *R* = 6 and the particle contact angle is θ_*p*_ = 46°. (b) Snapshot of droplet wetting at the particle surfaces. The substrate has a contact angle θ_*s*_ = 150° and the contact angle of particles is θ_*p*_ = 46°.

To understand this behavior, we show in Figure 6b the snapshot of drying a colloidal suspension film with θ_*p*_ = 46° after rupture on a substrate with a constant angle θ_*s*_ = 150°. The liquid wets at particle surfaces instead of the substrate, prohibiting the formation of a droplet and leading to a uniform, highly ordered deposit. We conclude that a lower particle contact angle eliminates the effect of substrate wettability on the deposition pattern.

### Theoretical Analysis

We propose a simple analytical analysis to predict the surface coverage fraction as a function of particle volume fraction. A thin film of particles with an initial thickness *z*_0_ and a particle volume fraction *c*_*v*_ is deposited on the substrate. The total volume of particles is *V*_*p*_ = *Sz*_0_ *c*_*v*_, where *S* is the surface of the film, equal to the area of the substrate. Assuming that all the particles are deposited on the substrate, we expect a surface coverage area as *S*_*p*_ = *N*_*p*_π*R* ^2^, where *N*_*p*_ is the total number of particles. With $N_{p}=\frac{V_{p}}{4/3\pi R^{3}}$ , we obtain *S*_*p*_ = 3*Sz*_0_ *c*_*v*_/4*R*. Note that the highest surface coverage of randomly placed and equally sized spheres in a 2D arrangement is about ϕ ∼ 0.77.^49^ It follows that the surface coverage fraction is given by

$$\phi =\frac{S_{p}}{S}=\{\begin{array}{ll} \frac{3z_{0}c_{v}}{4R} & \mathrm{if}\frac{3z_{0}c_{v}}{4R}<0.77\\ 0.77 & \mathrm{else} \end{array}$$ 23

Based on eq 23, we can draw the conclusion that with increasing the film thickness and particle volume fraction, a smaller particle radius leads to an increased particle surface coverage fraction. In Figure 3 we compare the analytical prediction eq 23 (solid lines) with our simulation results (symbols). For cases with lower substrate contact angles, eq 23 adequately captures the surface coverage fraction as a function of volume fraction. However, the model shows large deviations from the simulation results for a higher substrate contact angle. This can be attributed to particle clusters resulting from the formation of droplets.

To take into account this droplet formation, we assume that the rupture of the film is followed by a single colloidal suspension droplet being formed on the substrate with a higher contact angle. The droplet immediately reaches its equilibrium state and dries in a constant angle mode, leaving a spherical particle cluster on the substrate. We note that the contact angle of this droplet is determined by the particle contact angle if the particle contact angle is smaller than the substrate contact angle. The volume of this spherical particle cluster is

$$V=N_{p}\frac{4}{3}\pi R^{3}/\psi =Sz_{0}c_{v}/\psi$$ 24

in which ψ is the packing fraction of particles that is taken as the maximum random packing fraction of hard spheres ψ_max_ ≈ 60%. Assuming the particle cluster has a spherical cap shape with a contact angle of θ and a footprint of *a*, we can write its volume as

$$V=\frac{\pi }{6}\frac{1-\cos\;\theta }{\sin\;\theta }\left[3+{\left(\frac{1-\cos\theta }{\sin\theta }\right)}^{2}\right]a^{3}$$ 25

By combing eqs 24 and 25, we obtain the footprint of the deposit as

$$a={\left(\frac{z_{0}Sc_{v}}{\frac{\pi }{6}\psi_{\max}\frac{1-\cos\theta }{\sin\theta }\left[3+{\left(\frac{1-\cos\theta }{\sin\theta }\right)}^{2}\right]}\right)}^{1/3}$$ 26

where θ = min­(θ_*s*_, θ_*p*_). The surface coverage fraction is

$$\phi =\frac{S_{p}}{S}=\frac{\pi a^{2}}{S}$$ 27

In Figure 3, we compare eq 27 (dashed-dotted lines) with simulation results (symbols). The analytical prediction agrees well with the simulations for a particle contact angle θ_*p*_ = 90° and at lower volume fractions *c*_*v*_ < 0.13 on a substrate with θ_*s*_ = 150°. The deviation at higher volume fractions is likely due to the formation of multiple droplets following film rupture in the simulations, whereas our theory assumes the formation of a single droplet. We note that the droplet volume at rupture depends on the timing of the rupture event. After rupture, the droplet continues to evaporate, shrinking in a spherical cap, until reaching a critical volume where the particles achieve their maximum random packing fraction. Eq 24 describes this critical droplet volume with a maximal random packing fraction. Since the final particle cluster size is determined by this critical droplet volume, the droplet volume at the rupture moment does not directly influence our analysis or the results.

Our findings provide guidance for selecting appropriate solvents or substrates to form monolayers for particles with specific surface energies. The contact angle of the particles is determined by $\cos\;\theta_{p}=\frac{\gamma_{PG}-\gamma_{PL}}{\gamma_{LG}}$ and the contact angle of the substrate by $\cos\;\theta_{s}=\frac{\gamma_{SG}-\gamma_{SL}}{\gamma_{LG}}$ , where γ_*ij*_ represents the surface energy between component *i* and component *j* and *P*, *G*, *L*, *S* denote particle, gas, liquid, and substrate, respectively. It is preferable to choose a liquid with a lower surface energy γ_*LG*_ and a substrate with a higher surface energy γ_*SG*_. Regarding the optimal volume fraction for forming a monolayer with a maximal surface coverage fraction of 0.77 on a given substrate of area *S*, two cases are considered: (i) depositing a certain amount of solution on a substrate^15^: the solution volume is *V*_*d*_, then the optimal volume fraction is *c*_*v*_ = 1.027*RS*/*V*_*d*_; (ii) dip-coating or blade-coating at higher coating velocities^14,48^ : the coated film thickness *z*_0_ can be estimated using the Landau-Levich equation,^50,51^ and the optimal particle volume fraction is then *c*_*v*_ = 1.027*R*/*z*_0_ (based on eq 23).

## Conclusions

We numerically investigated the drying process of a colloidal suspension film on a substrate using a coupled lattice Boltzmann and discrete element method that fully resolves colloidal particles. This approach allows us to capture detailed information at the scale of individual particles (e.g., contact-line pinning), providing deeper insights into the deposition process as compared to existing theoretical models,^52–56^ which typically rely on convection-diffusion equations to govern the transport of colloidal particles.

We studied the drying dynamics of a colloidal suspension film and tracked the temporal evolution of the evaporated mass. Interestingly, we found that the assembled particle monolayer at the interface does not inhibit solvent evaporation. This is because solvent transfer occurs rapidly through the particle layer, creating a saturated region above the particles that does not affect the overall evaporation flux. The evolution of film thickness during the drying of a colloidal suspension closely resembles that of a pure liquid film, consistent with our theoretical analysis. Future work should focus on the transition when the aggregation of particle multilayers begins to affect the evaporation flux,^57^ which may lead to an improvement of theoretical models regarding skin formation in drying colloidal suspension droplets.^55,56,58^

Furthermore, we investigated the effect of substrate wettability and particle wettability on the deposition pattern. A substrate with low wettability repels the liquid, leading to the formation of droplets upon film rupture and promoting the accumulation of particles into clusters. In contrast, high substrate wettability facilitates better wetting and spreading of the liquid, resulting in more uniform deposition across the substrate surface. High substrate wettability proves favorable for the formation of a homogeneous monolayer. Moreover, it is commonly believed that a hydrophilic substrate is essential for forming highly ordered monolayers in drying a film.^13,22^ Surprisingly, our findings reveal that particles with high wettability can mitigate the influence of substrate wettability, as the liquid prefers to wet the particle surface instead of the substrate surface to reduce the total free energy. This facilitates the formation of highly ordered monolayers even on hydrophobic substrates. To support our simulations, we developed simple analytical models to predict the surface coverage fraction as a function of particle volume fraction, taking into account both particle and substrate wettability. The theoretical models, validated by simulation data, can be applied to predict the surface coverage fraction, potentially serving as a guide for selecting appropriate solvents or substrates to form monolayers of particles in experimental settings.

In this work, we focused on dilute suspensions of spherical particles and the formation of monolayers only. However, our methodology can be employed directly to investigate the deposition of multiple staggered layers,^59,60^ the effect of substrate edges^61^ or the impact of different particle shapes.^62,63^ Furthermore, our work can be extended to study the deposition of inks involving molecules and polymers used in catalysis,^64^ batteries^65^ and biomedical applications.^66^

## Data Availability

The data that support the findings of this study are openly available at 10.5281/zenodo.14620290.
